# Enhancing Target Detection: A Fluorescence-Based Streptavidin-Bead Displacement Assay

**DOI:** 10.3390/bios14100509

**Published:** 2024-10-17

**Authors:** Sireethorn Tungsirisurp, Nunzianda Frascione

**Affiliations:** Department of Analytical, Environmental & Forensic Sciences, Faculty of Life Sciences & Medicine, King’s College London, London SE1 9NH, UK; sireethorn.1.tungsirisurp@kcl.ac.uk

**Keywords:** biosensor, fluorescence-based, DNA aptamers, antisense strands

## Abstract

Fluorescence-based aptasensors have been regarded as innovative analytical tools for the detection and quantification of analytes in many fields, including medicine and therapeutics. Using DNA aptamers as the biosensor recognition component, conventional molecular beacon aptasensor designs utilise target-induced structural switches of the DNA aptamers to generate a measurable fluorescent signal. However, not all DNA aptamers undergo sufficient target-specific conformational changes for significant fluorescence measurements. Here, the use of complementary ‘antisense’ strands is proposed to enable fluorescence measurement through strand displacement upon target binding. Using a published target-specific DNA aptamer against the receptor binding domain of SARS-CoV-2, we designed a streptavidin-aptamer bead complex as a fluorescence displacement assay for target detection. The developed assay demonstrates a linear range from 50 to 800 nanomolar (nM) with a limit of detection calculated at 67.5 nM and a limit of quantification calculated at 204.5 nM. This provides a ‘fit-for-purpose’ model assay for the detection and quantification of any target of interest by adapting and functionalising a suitable target-specific DNA aptamer and its complementary antisense strand.

## 1. Introduction

Aptamers are short, single-stranded oligonucleotides designed to have a specific binding interaction with the target of interest [1]. Aptamers are selected through a process called systematic evolution of ligands by exponential enrichment (SELEX), and their target binding properties are characterised using several different techniques [2,3]. Target binding affinities of aptamers can vary from nanomolar to micromolar range, often comparable to their antibody counterparts [1,2]. They can be used as the recognition moiety of a biosensor. In the last decade, aptamers have become popular among biosensor design due to their wide range of applications, ease of synthesis at low cost, and ability to undergo chemical conjugation and sequence manipulation without compromising target binding properties [1,3,4]. Due to their use in various sensing platforms, aptamers have been adopted in many fields, including medical, therapeutic, environmental, and diagnostic applications [2,4,5,6,7,8,9,10,11].

Aptamer-based biosensors, or aptasensors, are devices designed with an aptamer as the biological recognition component capable of producing a measurable signal upon target-specific interactions. To date, numerous aptasensors have been developed for the detection and quantification of analytes ranging from small toxins to larger proteins [10,12,13,14,15,16,17,18] and implemented into various assays ranging from colourimetric immunoassays for medical applications [19] to lateral flow assays (LFAs) for diagnostic applications [20,21,22]. The binding of an aptamer to its target can be transduced into different measurable signals (i.e., optical, colourimetric, and electrochemical) [9,12,13,23,24,25]. Colourimetric transducers, including LFAs, offer simple and fast target detection with very little instrumentation needed [20,22,26]. However, the assays are often only qualitative or semi-quantitative [20,22,27]. While an electrochemical transducer is excellent for small molecule detection due to its high sensitivity [13,16,22,27,28,29], it requires intricate and costly instrumentation [9,30,31]. Fluorescence-based optical aptasensors offer several advantages due to their sensitivity and simplicity over other signalling outputs. Fluorescence aptasensors are often designed with one or more fluorescent reporters that can be detected upon interaction with the target of interest.

While there are many mechanisms by which fluorescence detection can be achieved, some take advantage of the target-induced, structure-switching properties of DNA aptamers. Molecular beacons utilise the conformational change of DNA aptamers from a hairpin structure to produce a fluorescence change [5,17,18,32,33]. This fluorescence change, however, is based on a 1:1 stoichiometric binding of an aptamer to the target. In addition, not all the developed DNA aptamers undergo a complete dose-dependent structural switch [34]. Moreover, some DNA aptamers have been reported to have a high rigidity in the DNA structure, potentially hindering the conformational change upon target interaction [35,36]. This often leads to further design and optimisation of an appropriate loop-stem hybridisation probe to allow fluorescence change upon target-induced conformational change [37]. Alternatively, the use of complementary ‘antisense’ strands allows for a fluorescence change to be detected through a target-specific displacement of the antisense strands due to the competition for binding with the target [12,23,24,25,37]. This complete detachment of the antisense strands produces higher detectable signals, resulting in an improved assay sensitivity, as demonstrated in the previously reported anti-ATP electrochemical aptasensor assay [38]. Recent studies also implemented the use of mismatch antisense strands as an alternative to full match antisense to improve sensitivity towards a target analyte by increasing structural elasticity and weakening the aptamer—antisense bonding strength [30,38].

**Figure 1:**
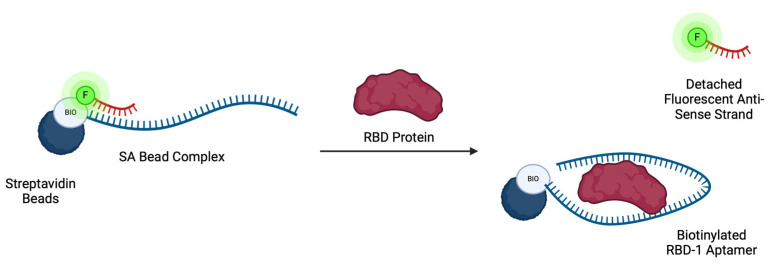
Schematic illustration of a fluorescence-based SA bead displacement assay. A DNA aptamer is initially hybridised with a complementary fluorescently labelled antisense strand to form a duplex. The duplex is then immobilised onto streptavidin-coated agarose beads, forming a SA bead complex. Upon addition of the target protein, the immobilised aptamer sequence preferentially binds to the target, displacing the fluorescently labelled strand. Displaced strands can then be collected and quantified.

In this work, the design and use of a streptavidin-aptamer (SA) bead complex as a fluorescence displacement assay for target detection is reported. A target-specific aptamer is hybridised with a suitable, fluorescently labelled antisense strand, and the duplex is subsequently functionalised onto a streptavidin-coated agarose resin solid support (Figure 1). To evaluate the applicability of this design, a DNA aptamer against the receptor binding domain (RBD) of SARS-CoV-2 was used as a model aptamer, having been previously characterised through binding affinity studies and target-binding simulations [36,39]. The SARS-CoV-2 virus is a member of the coronaviruses responsible for the COVID-19 global pandemic [40]. The RBD-1 aptamer was selected through SELEX [39] against the RBD located within the spike (S) proteins on the surface of the coronavirus. Sequence modifications allowed the selected aptamer to reach optimal target binding affinity with a reported K_D_ value of 5.8 nM [39].

To develop and validate this SA bead displacement assay, the antisense strand sequence, complex assembly protocol, and incubation times were optimised, and its applicability to the detection and quantification of the RBD protein was evaluated.

## 2. Materials and Methods

### 2.1. Material and Reagents

A DNA aptamer specific to RBD protein (RBD-1) [39] (5′-CAGCACCGACCTTGTGCTTTGGGAGTGCTGGTCCAAGGGCGTTAATGGACA-3′) was synthesised with a biotin labelled on the 5′-end, desalted, and lyophilised by Merck Life Science (Darmstadt, Germany). Antisense strands (sequences as shown in Table S1) were synthesised with either Cy5 or FAM fluorophores conjugated to the 5′-end by Merck Life Science (Darmstadt, Germany). Recombinant receptor binding domain (RBD) subunit protein was purchased from Cambridge Bioscience (Cambridge, UK).

For the enzyme-linked oligonucleotide assay, Nunc MaxiSorp™ clear, flat-bottom 96-well plates, streptavidin-conjugated horseradish peroxidase (SA-HRP), 3,3′,5′-tetramethylbenzidine (TMB) substrate solution, and 1 M sulphuric acid solution were obtained from Thermo Fisher Scientific (San Jose, CA, USA). Bovine serum albumin (BSA) was purchased from Merck Life Science (Darmstadt, Germany). All buffers were made in-house prior to the experiments. All absorbance measurements were performed using an Opsys MR UV-Vis microplate reader (Dynex Technologies, Chantilly, VA, USA) at 450 nm.

For the SA bead displacement assay, white, flat-bottom 96-well plates and Pierce™ NeutrAvidin™ Agarose beads (SA beads) were purchased from Thermo Fisher Scientific, while Spin-X centrifuge Filters (Corning Costar) were purchased from Fisher Scientific (San Jose, CA, USA). Assay buffer (137 mM NaCl, 2.7 mM KCl, 10 mM Na_2_HPO_4_, 1.8 mM KH_2_PO_4_, 10 mM MgCl_2_ at pH 7.3) was prepared in-house. All fluorescence measurements were performed on the Agilent Cary Eclipse Fluorescence Spectrophotometer (Santa Clara, CA, USA) with a microplate reader accessory. For the FAM-conjugated antisense strands, the fluorescence was measured at Ex/Em 495/520 nm, while for the Cy5-conjugated antisense strands, the fluorescence was measured at Ex/Em 651/674 nm.

### 2.2. Enzyme-Linked Oligonucleotide Assay (ELONA)

Prior to aptamer incubation, a serial dilution of RBD-1 was prepared in binding buffer (BB; PBS, 0.55 mM MgCl_2_, 1% BSA, 0.05% Tween-20) with concentrations ranging from 1.56 nM to 100 nM. A stock solution of RBD protein was prepared to 0.5 µg/mL in carbonate/bicarbonate coating buffer pH 9.6, and 100 µL of RBD solution was added to each well and incubated overnight at 4 °C. After immobilisation, the wells were washed three times with washing buffer (WB; PBS, 0.55 mM MgCl_2_, 0.05% Tween-20). The wells were blocked by incubating 200 µL of blocking buffer (PBS, 0.55 mM MgCl_2_, 1% BSA) for 1 hr at room temperature (RT) with shaking. After washing three times with WB, 100 µL of different aptamer solutions were incubated in the corresponding wells for 1 h at RT with shaking. The wells were washed three times with WB to remove any unbound aptamers before 100 µL of 1 µg/mL of SA-HRP was incubated for 45 min at RT with shaking. Lastly, following washing with 3 × WB, 100 µL of TMB substrate solution was added and incubated for 20 min at RT with shaking. After 20 min, the oxidative reaction was stopped by adding 100 µL of 1 M sulphuric acid. The absorbance measurements were performed immediately using the microplate reader at 450 nm. Any background absorbance from the negative control was subtracted from the sample readings for RBD-specific binding analysis.

### 2.3. Assembly of Streptavidin-Aptamer (SA) Bead Complex

Prior to assembly with SA beads, an RBD-1 aptamer solution was equilibrated and hybridised to the corresponding antisense strands. A stock solution of RBD-1 aptamer and each antisense strand were mixed in assay buffer at concentrations of 2.5 µM and 2.75 µM, respectively. The mixtures were denatured at 99 °C for 3 min before lowering to an annealing temperature of 60.9 °C at 0.1 °C/sec ramp rate. The mixtures were incubated at the calculated annealing temperatures for 10 min before slowly cooling down to 10 °C until use. The aptamer—antisense duplex solutions were adjusted to 250 µL final volume in assay buffer with 0.5 µM and 0.55 µM working concentrations of aptamer and antisense strand, respectively.

The SA beads were pre-conditioned prior to aptamer functionalisation by adding 100 µL of resuspended SA beads to Spin-X centrifuge filters, and the filter unit was centrifuged at 500× *g* for 1 min to remove the storage buffer. To remove any labile streptavidin molecules on the bead surface, SA beads were incubated with 250 µL of 200 mM NaOH for 30 min at RT with shaking. The SA beads were washed five times with 250 µL PBS with centrifugation under the same conditions. To assemble the SA bead complex, 250 µL of hybridised aptamer—antisense duplex solution was added to the washed SA beads and incubated for 30 min at RT with shaking. The complex was washed twice with 250 µL assay buffer in the same condition, ready for further experiment.

### 2.4. Time-Point Optimisation

The prepared SA bead complex was incubated in assay buffer at RT with shaking at different time points (i.e., 15, 30, 45, 60, 90, and 120 min). For each time point, the complex was centrifuged at 500× *g* for 1 min, and the fluorescence measurement was performed on the flowthrough to evaluate the non-specific displacement of antisense strands.

### 2.5. RBD Detection and Quantification

For the qualitative assessment of displacement, an RBD solution was prepared in 250 µL assay buffer at a concentration of 4000 nM. For the quantitative displacement assessment, different concentrations of RBD solution were prepared in 250 µL assay buffer ranging from 50 nM to 2000 nM. The RBD solutions were added to the prepared SA bead complex and incubated for 15 min at RT with shaking. To collect any displaced antisense strands, the complex was centrifuged at 500× *g* for 1 min, and the flowthrough was subjected to fluorescence measurement using an Agilent Cary Eclipse fluorescence spectrophotometer.

## 3. Results and Discussion

### 3.1. Target Binding Affinity and Specificity

ELONA is a microplate-based technique that uses aptamers, offering performance and high throughput comparable to ELISA. Therefore, the technique was chosen to confirm RBD-1 aptamer target binding affinity and specificity. The concentration of RBD was optimised to 0.5 µg/mL for immobilisation on the surface of the ELONA plate. The RBD-1 aptamer used for the incubation was biotin-modified on the 5′-end. Serial dilutions of the RBD-1 aptamer (from 1.5625 nM to 100 nM) were incubated with the immobilised RBD for an hour with shaking. A negative control (i.e., binding buffer with no RBD-1 aptamer) was included. SA-HRP was then conjugated to the bound aptamers and used as the catalyst for a colourimetric assay with TMB substrate. Absorbance was measured using UV-Vis spectroscopy. The absorbance readings were plotted against the concentrations of the RBD-1 aptamer, and a dose-response curve was generated.

**Figure 2:**
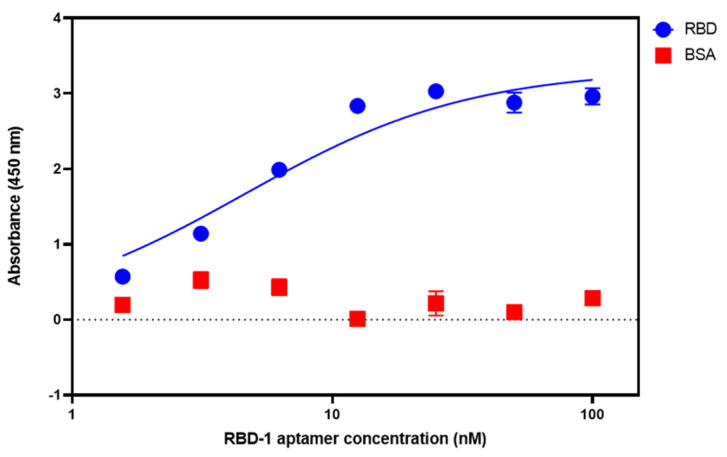
Simulated dose response curve of RBD-1 aptamer showing high binding affinity to RBD protein (blue). No binding was observed when the same aptamer sequence was incubated with BSA (red). The K_D_ value was estimated at 4.58 ± 1.15 nM using a non-linear regression one-site specific binding model on GraphPad Prism. (Error bar = s.d., n = 3).

From the ELONA dose-response curve data (Figure 2), the RBD-1 aptamer showed high RBD binding affinity with a dissociation constant (K_D_) of 4.58 nM, comparable to the K_D_ value reported in the literature [39]. The RBD-1 aptamer was also tested for binding specificity by incubation with bovine serum albumin (BSA) immobilised at 0.5 µg/mL on the ELONA plate. Minimal absorbance was observed, suggesting no cross-reactivity with BSA (Figure 2).

### 3.2. Assembly of the Streptavidin-Aptamer (SA) Bead Complex

The success of this type of assay relies on an appropriate complex assembly method that allows a sufficient amount of aptamer and the reporting antisense strands to be loaded onto the SA beads. To achieve this, the RBD-1 aptamer was initially denatured and hybridised with the reporting antisense strand using a standard thermocycler. This was to ensure that the RBD-1 aptamer was complemented with the antisense strand prior to loading onto the SA beads. After mixing the aptamer with the corresponding antisense strands in the assay buffer, the solution was heated to 99 °C for 3 min to denature any secondary structures within RBD-1 and antisense strands that could hinder hybridisation. The temperature was then lowered to the annealing temperature at 0.1 °C/sec ramp rate. The slow decline in temperature allows hybridisation to take place. The annealing temperature was calculated using the equation

$$T_{a}=0.3\left(T_{as}\right)\times 0.7\left(T_{apt}\right)-14.9$$

where *T_a_* is the annealing temperature, *T_as_* is the melting temperature of the corresponding antisense strand, and *T_apt_* is the melting temperature of the RBD-1 aptamer [41].

After incubating at the annealing temperature for 10 min, the solution was cooled down to 10 °C until use. This kept the hybridised duplex stable until the streptavidin beads were ready for assembly and use for target detection.

**Figure 3:**
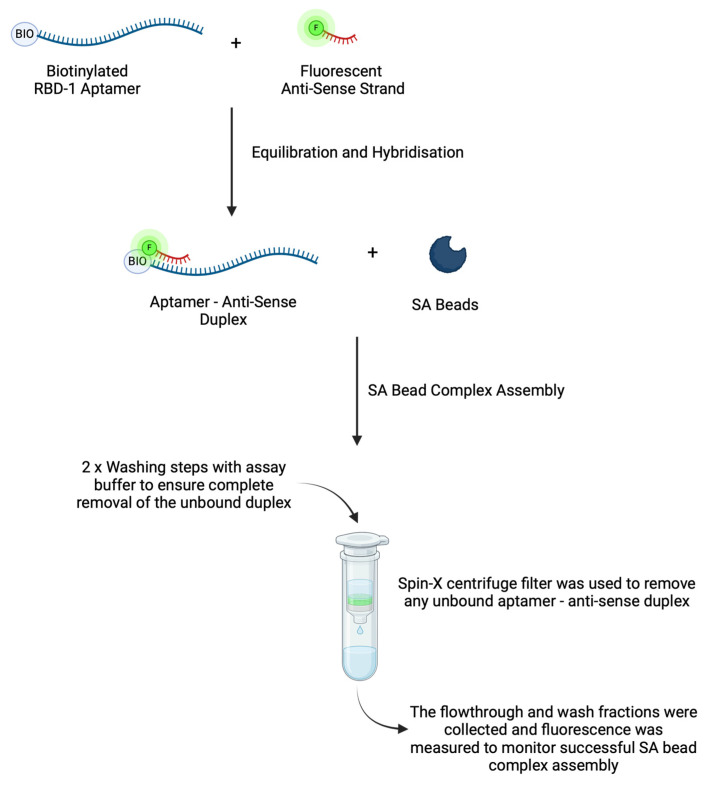
Schematic illustration of SA bead complex assembly. Initially, a mixture of biotinylated RBD-1 aptamer and fluorescently labelled antisense strands was equilibrated and hybridised at the calculated annealing temperature. Subsequently, the aptamer—antisense duplex was incubated with pre-washed SA beads to form the SA bead complexes. A washing step was then performed to remove unbound aptamer—antisense strand duplexes from the complex solution. The SA bead complexes were then stored in assay buffer until use.

The streptavidin beads were prepared and conditioned prior to functionalisation with the aptamer—antisense duplex. The amount of beads used was calculated according to the manufacturer’s recommendations and based on the bead capacity, while the beads conditioning procedure was performed according to the manufacturer’s protocol [42]. After removing the storage buffer via centrifugation, a strong basic solution was used to remove any labile streptavidin molecules that could interfere with the functionalisation. Subsequent extensive washes ensured no residual NaOH remained in the bead suspension that could affect the functionalisation of the aptamer—antisense duplex. To assemble the SA bead complex (Figure 3), the aptamer—antisense duplex solution was added to the washed SA beads. The complex was washed to remove any unbound duplexes and stored in assay buffer until use. The washing steps were implemented here to ensure the complete removal of unbound aptamer—antisense duplexes from the SA bead complexes. Any unbound duplex left in the complex solution can interfere with the downstream target binding and fluorescence measurement. While these additional washing steps might be considered impractical compared to homogeneous mix-and-read formats, this displacement assay offers an alternative aptasensing platform where a traditional molecular beacon might not be suitable or higher sensitivity is required.

**Figure 4:**
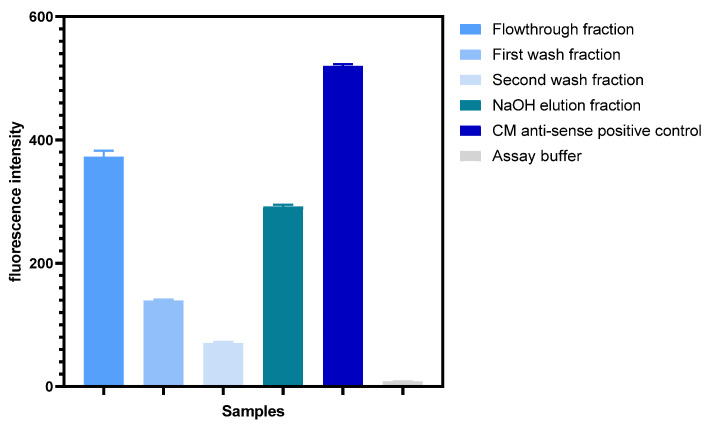
Fluorescence measurements of the collected fractions from SA bead complex assembly. The fluorescence measured from flowthrough and washes corresponded to the removed unbound aptamer—antisense duplexes from the complex. The high fluorescence intensity shown in the NaOH elution fraction suggested that the bound antisense strands were detached from the complex and, therefore, that the SA bead complex assembly was successful. (Error bar = s.d., n = 3).

Spectrophotometry was used to evaluate the success of SA bead complex assembly. To investigate the amount of aptamer—antisense duplex conjugated to the SA bead surface, the assembled complex was incubated with 200 mM NaOH for 30 min, and the denatured bound antisense strands were collected. As the aptamer—antisense duplex is in 1:1 stoichiometry, the amount of antisense strands is proportional to the amount of RBD-1 aptamer available on the SA bead complex for target binding. Fluorescence measurements were performed on the collected fractions (Figure 4). A substantial fluorescence signal was observed in the flowthrough fraction, suggesting that a portion of the aptamer—antisense duplexes did not bind to the beads during assembly. This could be due to the limited surface area of the beads hindering functionalisation capacity. Each washing step allowed the separation of the residual unbound duplexes from the complex solution. The low fluorescence intensity observed from both washes indicated that the unbound duplexes had been removed. To investigate the amount of aptamer—antisense duplexes functionalised on the beads, the washed complex was incubated with a basic solution to denature the fluorescently labelled antisense strands for quantification. A high fluorescence intensity was observed in the NaOH elution fraction (Figure 4), indicating the successful functionalisation of the aptamer—antisense duplexes on the streptavidin beads. This assembly method has proven successful for further assay evaluation. Using a Cy5 calibration curve (Figure S1), it was estimated that approximately 150 nM of antisense strands, hence 150 nM of RBD-1 aptamer, were functionalised on the SA bead complex. This equates to approximately 37.5 pmoles of the aptamer—antisense duplex per 100 µL of the bead resin.

### 3.3. Incubation Time Optimisation

The stability of the aptamer—antisense duplex is unknown; therefore, it is important to investigate a time range within which the duplex remains stable within the complex for target detection and quantification. Hence, an incubation time assay was performed to investigate any non-specific displacement of the antisense strands. The assembled SA bead complex was incubated with assay buffer at RT for a time ranging from 15 to 120 min. After incubation at each time point, the complex was centrifuged using a Spin-X centrifuge filter, and the fluorescence measurement was performed on the collected fractions to check for the detached antisense strands.

**Figure 5:**
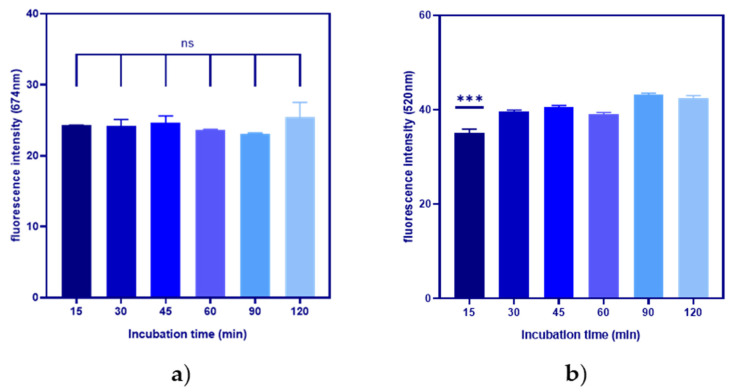
Timepoint optimisation to evaluate the suitable incubation time of the SA bead complex without significant non-specific displacement using (**a**) CM and (**b**) MM-T9 antisense strand. While CM shows no significant difference between each time point, MM-T9 demonstrated that incubations longer than 15 min resulted in significant non-specific displacements. (Error bar = s.d., n = 3, ns = not significant, *** = *p* < 0.001).

For this experiment, two different fluorescently labelled antisense strands were used, namely a complete match (CM) and a mismatch (MM-T9) strands, to evaluate the effect of the different antisense sequences on the non-specific displacement from the aptamer strand. MM-T9 contains a T→A mutation at the 9th nucleotide, while CM is a full-match complementary strand. A one-way ANOVA test was used to evaluate the significance of variations between the time points to check whether the incubation time influenced non-specific displacement and, if so, identify the suitable incubation time for the SA bead displacement assay. The CM antisense strand provided a stable SA bead complex with minimal non-specific displacement up to the two-hour time point (Figure 5a). In contrast, significant non-specific displacement of MM-T9 was observed with incubations longer than 15 min (Figure 5b). This suggests that the single mutation within the antisense strand had a detrimental effect on the hybridisation affinity to the RBD-1 aptamer. This could also indicate that, for target detection applications, the SA bead complex using MM-T9 would need to be used shortly after complex assembly. An incubation time of 15 min was therefore identified to be the optimal target incubation time for the SA bead complex.

### 3.4. RBD Protein Detection and Quantification

Initially, different antisense strands (Table S1) were investigated for their RBD-induced displacement capability. The SA bead complexes with the corresponding aptamer—antisense duplexes were incubated with 4000 nM RBD protein solution. After a 15 min incubation, each complex was centrifuged using a Spin-X centrifuge filter, and the fluorescence of the collected fractions was measured (Figure S2). Based on the preliminary RBD-induced displacement experiment (Figure S2), three antisense strands were chosen for further optimisation (i.e., CM, MM-T2, and MM-T9).

The hybridisation and detachment upon target binding of complete match (CM) antisense were evaluated and compared to mismatch (MM) antisense strands. As mentioned earlier, antisense strands were implemented in this study to potentially overcome the rigidity of the RBD-1 aptamer structure [36] and to enhance the response signal and assay sensitivity. Most reported studies on DNA aptamer—antisense duplexes utilised CM antisense strands [23,24,31]. However, CM antisense strands can, at times, form very strong duplexes with DNA aptamers, prevent displacement and interfere with the target binding interactions [38,43]. Therefore, MM antisense strands were implemented as alternative duplex constructions to improve signal measurement and assay sensitivity [30,38].

**Figure 6:**
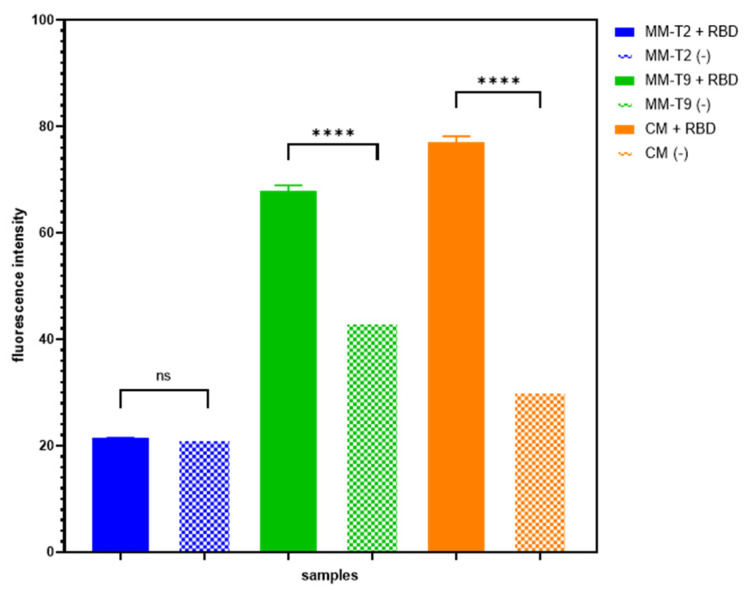
Qualitative analysis of SA bead complex using various antisense strands. SA bead complexes with three different antisense strands, including CM (orange), MM-T2 (blue) and MM-T9 (green), were tested by incubating the complexes with 2000 nM RBD (+RBD) or assay buffer (-). Only CM and MM-T9 antisense strands showed significant RBD-specific displacements. (Error bar = s.d., n = 3, ns = not significant, **** = *p* < 0.0001).

Therefore, SA bead complexes with the abovementioned antisense strands were assembled and incubated with either assay buffer or 4000 nM RBD solution to determine a potential RBD-specific displacement of each antisense (Figure 6). A one-way ANOVA test was used to evaluate the difference between non-specific (-) and RBD-specific (+RBD) displacements of each antisense strand. Only CM and MM-T9 antisense strands demonstrated a significant difference (*p* < 0.0001) between the (-) and (+RBD) samples and, therefore, were selected for further quantitative analysis. Interestingly, the (-) control samples of the MM-T9 antisense are higher compared to other antisense strands (-) samples. This could suggest a substantial non-specific displacement of the MM-T9 antisense duplexes.

**Figure 7:**
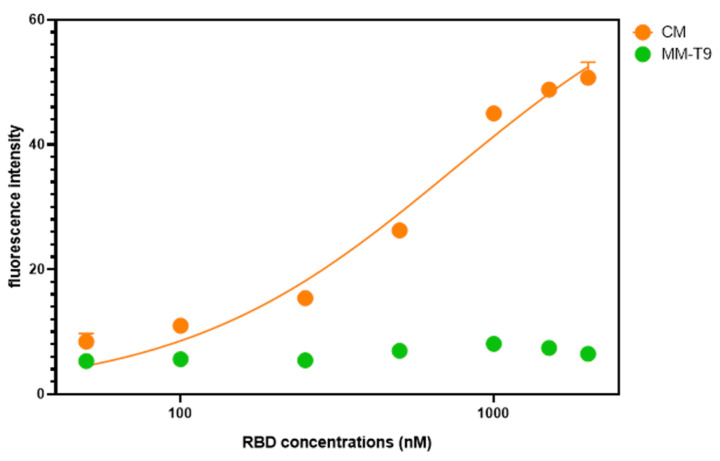
SA bead displacement assay with CM (orange) demonstrated RBD-specific dose-dependent fluorescence response. Meanwhile, MM-T9 (green) did not show an RBD-dependent detachment from the SA bead complex. This suggests the CM antisense strand is an appropriate antisense strand of the SA bead complex assembly for the SA bead displacement assay. (Error bar = s.d., n = 3).

To evaluate the dose-dependent displacement of the CM and MM-T9 antisense strands, different concentrations of RBD solution were prepared in assay buffer (50 nM, 100 nM, 250 nM, 500 nM, 1000 nM, 1500 nM, and 2000 nM). Following a 15 min incubation of the SA bead complex with different RBD solutions, the complex was centrifuged, and the fluorescence of the collected flowthroughs was measured. The fluorescence readings were plotted against the incubated RBD concentrations (Figure 7). The CM antisense strand demonstrated a good RBD-specific dose-dependent displacement (Figure 6; orange curve), while MM-T9 did not produce a response in the presence of RBD even at the highest concentration of 2000 nM. Therefore, it was concluded that the displacement observed within Figure 5 was substantial non-specific displacement of the MM-T9 antisense strands. Hence, this explains why MM-T9 failed to produce a dose-dependent response (Figure 7). This further confirmed that CM was the most suitable antisense strand to use as part of the SA bead complex for the developed displacement assay, as dose-dependent displacement was observed in the range of 50 nM—2000 nM.

### 3.5. Assay Sensitivity

**Figure 8:**
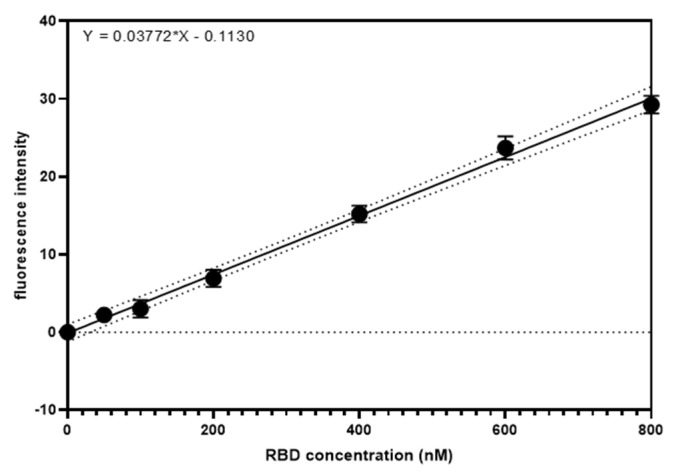
A linear curve of RBD-specific dose-dependent fluorescence increase. A good linear range is observed between 50 and 800 nM RBD with an R-squared of 0.9962, suggesting a good linear fit. (Error bar = s.d., n = 3). (Solid line: linear regression line; dotted line: 99% confidence intervals; Black bullet: experimental mean values).

Under the optimised experimental conditions, RBD-1 aptamer (500 nM) and the CM antisense strand (550 nM) were hybridised, and the duplex was functionalised on streptavidin beads for the detection of RBD protein in solution. For the protein quantification, the assay demonstrated a good linear range between 50 nM and 800 nM of RBD (Figure 8).

A linear regression model was used to generate the RBD quantification equation as

$$Y=37.72\left(X\right)-0.1130$$

with an R-squared value of 0.9962, demonstrating a good data fitting. The limit of detection (LOD) was calculated at 67.5 nM using 3.3σ/S, where σ is the standard deviation of the response, and S is the slope of the linear curve (Table 1). Meanwhile, the limit of quantification (LOQ) was calculated at 204.5 nM using 10σ/S (Table 1). While this might not be as sensitive as other developed aptasensors, including electrochemical or lateral flow assays [37], this optical displacement assay has shown to be a selective and rapid method for RBD protein detection. Moreover, this model SA bead displacement assay is highly adaptable for application with any target of interest by functionalisation of target-specific aptamers and their corresponding antisense strand. The relative standard deviation (RSD%) values of the SA bead displacement assay were calculated to be between 3.7 and 8.7%, indicating an effective RBD measurement using the developed method. The RSD% values were comparable to other aptamer-based sensors previously developed [44,45,46,47] and were compliant with the guidelines for bioanalytical method validation [48].

## 4. Conclusions

In this study, we demonstrated that a streptavidin-aptamer (SA) bead complex can be developed as a fluorescence-based aptasensor for the detection and quantification of the SARS-CoV-2 RBD protein in solution. For the first time, streptavidin beads were integrated as part of a fluorescence displacement assay for the detection and quantification of the target analyte of interest. The beads provide solid support for the assay system for both complex preparation and target detection. For the complex preparation, the solid support of the beads allows the removal of unbound aptamer—antisense duplexes and reduces background fluorescence. Meanwhile, this solid support allows the separation of the detached antisense strands from the complex during target detection, resulting in improved sensitivity. The RBD-1 aptamer was chosen as the model aptamer and demonstrated adequate target binding affinity and specificity, as shown by ELONA (K_D_ value estimated at 4.58 nM). The CM antisense strand was hybridised onto the RBD-1 aptamer before functionalising the duplex onto the preconditioned streptavidin beads. In the presence of the RBD protein, the CM antisense strand was displaced from the complex, and a fluorescence signal could be measured. A good linear fluorescence increase was recorded between 50 and 800 nM RBD. The LOD of this displacement assay was calculated at 67.5 nM, while the LOQ was calculated at 204.5 nM. Compared to other developed optical sensors, this assay is considered suitable for detecting and quantifying RBD protein. While the amount of RBD protein present in SARS-CoV-2 is unknown, this developed assay could be implemented to detect and quantify the SARS-CoV-2 virus in future works. Most importantly, this model assay demonstrates adaptable and versatile applications for target detections and quantifications of any target of interest by functionalising a suitable target-specific DNA aptamer and a complementary antisense strand to create a ‘fit-for-purpose’ SA bead complex.

## Figures and Tables

**Table 1:** Simulated parameters from linear regression model generated using GraphPad Prism.

Parameters	Values
Equation	Y = 0.03772(X) − 0.1130
Slope	0.03772
R-squared	0.9962
Sy.x (σ)	0.7715
LOD	67.5 nM
LOQ	204.5 nM
RSD%	3.7–8.7%

## Data Availability

Data is contained within the article or Supplementary Materials.

## Supplementary Materials

The following supporting information can be downloaded at: https://www.mdpi.com/article/10.3390/bios14100509/s1. Figure S1. Calibration curve of Cy5-CM for the quantification of displaced Cy5-CM in SA bead displacement assay. Figure S2. Evaluation of suitable antisense strands for SA bead displacement assay. Table S1. A summary list of antisense strands used for optimisation and their primary sequences.
