# Supplementary material for: Enhancing Target Detection: A Fluorescence-Based Streptavidin-Bead Displacement Assay
Source: Biosensors (Basel). 2024 Oct 17;14(10):509. doi: 10.3390/bios14100509 (PMC12365907; doi:10.3390/bios14100509)
Supplement: Supplementary file 1 [file biosensors-14-00509-s001.zip › biosensors-3238462-supplementary.pdf]

biosensors

MDPI

Supplementary Materials

Enhancing Target Detection: A Fluorescence-Based Streptavidin-Bead Displacement Assay

Sireethorn Tungsirisurp and Nunzianda Frascione *

Department of Analytical, Environmental & Forensic Sciences, Faculty of Life Sciences & Medicine, King's College London, London SsE1 9NH, UK; sireethorn.1.tungsirisurp@kcl.ac.uk
* Correspondence: nunzianda.frascione@kcl.ac.uk; Tel.: +44(0)-20-7848-3842

Cy5 calibration curve for CM antisense quantification

Material and method

A serial dilution of Cy5-MM antisense strand was prepared with concentrations ranging from 6.25 nM to 250 nM in assay buffer (PBS, 10 mM $MgCl_2$). To measure the fluorescence intensity of each concentration, 100 μL of each Cy5-MM solution was added to a white, flat-bottom 96-well plate and measured under a fluorescence spectrophotometer. The fluorescence intensity was plotted against the Cy5-MM concentration, and a linear regression was analysed using GraphPad Prism.

Results

For antisense strand quantification, the assay demonstrated a good linear range between 6.25 nM and 250 nM of Cy5-CM antisense (Figure S1). A linear regression model was used to generate a linear equation for Cy5-CM quantification to be

$$Y = 0.7142(X) - 2.481$$

with an R-squared of 0.9962, demonstrating a good data fitting. The limit of detection (LOD) was calculated at 17.5 nM, and the limit of quantification (LOQ) was calculated at 53 nM.

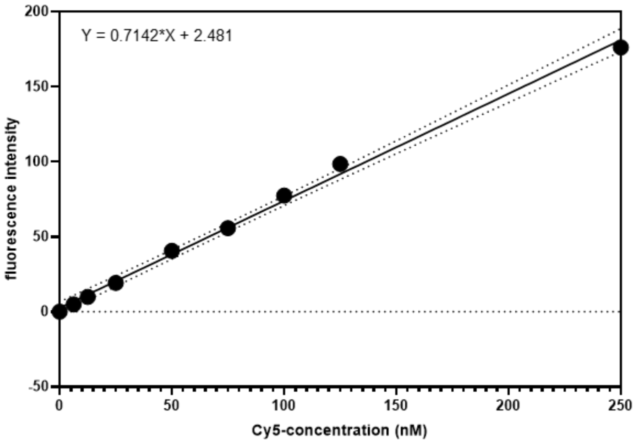

Figure S1. Calibration curve of Cy5-CM for the quantification of displaced Cy5-CM in SA bead displacement assay. A linear regression model was simulated using GraphPad Prism. (Solid line: linear regression line; dotted line: 99% confidence intervals; Black bullet: experimental mean values).

Table S1. A summary list of antisense strands used for optimisation and their primary sequences.

Names	Sequences (5′ → 3′)	Bases
CM	Cy5—GTCGGTGCTG	10
MM-T2	FAM—G**A**CGGTGCTG	10
MM-T6	FAM—GTCGG**A**GCTG	10
MM-T9	FAM—GTCGGTGC**A**G	10
MM-T2T6	FAM—G**A**CGG**A**GCTG	10
MM-T2T9	FAM—G**A**CGGTGC**A**G	10
MM-T6T9	FAM—GTCGG**A**GC**A**G	10

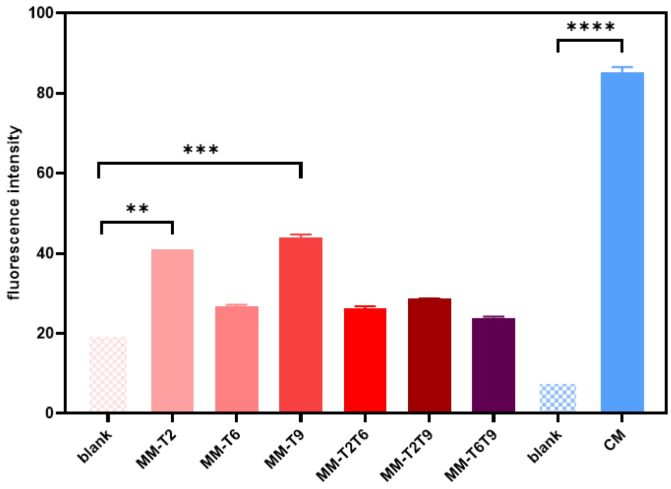

Figure S2. Evaluation of suitable antisense strands for SA bead displacement assay. Three antisense strands, namely CM, MM-T2, and MM-T9, showed various degrees of significant displacement upon incubation with RBD, whereas the other four strands did not show significant displacement. Only the two strands that showed the most significant displacement were selected for future analysis (MM-T9 and CM). (** = $P \leq 0.01$, *** = $P \leq 0.001$, **** = $P \leq 0.0001$).
